# Highly expressed FAM189B predicts poor prognosis in hepatocellular carcinoma

**DOI:** 10.3389/pore.2022.1610674

**Published:** 2022-11-25

**Authors:** Wanshan Ma, Xiaoning Zhang, Chenchen Ma, Peng Liu

**Affiliations:** ^1^ Department of Clinical Laboratory Medicine, The First Affiliated Hospital of Shandong First Medical University & Shandong Provincial Qianfoshan Hospital, Shandong Medicine and Health Key Laboratory of Laboratory Medicine, Jinan, Shandong, China; ^2^ Central Laboratory, Affiliated Hospital of Shandong University of Chinese Traditional Medicine, Jinan, Shandong, China

**Keywords:** biomarker, prognosis, hepatocellular carcinoma, clinical significance, FAM189B

## Abstract

Hepatocellular carcinoma (HCC) is one of the most malignant tumors with persistently high morbidity and mortality. However, the expression, prognostic and clinical significance of FAM189 family genes in HCC remain largely unknown. In this study, the expression levels of FAM189 family genes in HCC were analyzed through TCGA-LIHC and ICGC-LIRI-JP cohorts, and further validated in multiple independent GEO datasets. It was found that the expression of FAM189B was significantly upregulated in HCC tumor tissues, while the expression of FAM189A1 and FAM189A2 was not significantly changed between tumor and adjacent tissues. Further analysis revealed that upregulated copy number variation contributed to increased expression of FAM189B in HCC. Survival analysis showed that highly expressed FAM189B was significantly correlated with unfavorable prognosis, including overall survival, disease-specific survival, and progression-free interval. Univariate and multivariate Cox regression analysis showed that FAM189B was a potential novel prognosis factor for HCC patients. In addition, the association between FAM189B expression and clinical and molecular characteristics was analyzed. High expression of FAM189B was associated with high AFP level, high predicted risk metastasis signature, and TP53 mutation, while there was no significant association between FAM189B expression and cancer stage or tumor grade of HCC. Gene set enrichment analysis revealed that highly expressed FAM189B was closely related with signal pathways and biological processes associated with cell proliferation and cell cycle in HCC. In conclusion, this study suggested that FAM189B was highly expressed in HCC and highly expressed FAM189B may serve as an effective prognostic indicator and a potential therapeutic target for HCC patients.

## Introduction

Hepatocellular carcinoma (HCC) is a serious malignancy with persistently high morbidity and mortality, causing more than 500,000 deaths every year and ranking the fourth most common cause of cancer related death in the world (1, 2). HCC usually evolves from chronic hepatitis B or C virus infection, excessive alcohol consumption, metabolic liver disease, and exposure to aflatoxins and aristolochic acid (3–5). Over the past decades, majority of HCC patients were still detected at an advanced stage, and incidence and cancer-specific death still continued to increase in many countries in the world (6, 7). Serum alpha-fetoprotein (AFP) assay is a traditional useful method for diagnosis and monitoring of HCC, but the application of AFP for liver cancer is unsatisfactory, and it is not sufficient to predict the prognosis for HCC patients effectively (8). Further, despite some achievements in HCC biomarker discovery, the prognosis for HCC patients remains unsatisfactory, with a 5-year survival rate less than 30%. Therefore, there is still an urgent need to identify new effective biomarkers and therapeutic targets.

FAM189 (family with sequence similarity 189) family consists of three protein-coding members including FAM189A1, FAM189A2 and FAM189B, which are paralog genes of each other. FAM189A1 is a CD20-like multiple-pass transmembrane protein that is required for cell signaling (9). Aberrant methylation level of CpG islands related to FAM189A1 was associated with attenuated sperm motility in males suffering from reduced fecundity (10). FAM189A2 encodes a type-Ⅰ transmembrane protein which is primarily expressed at the plasma membrane (11). The expression of FAM189A2 was downregulated in breast cancer and low expression of FAM189A2 was significantly associated with a reduction in the relapse free survival (12). In oral squamous cell carcinoma, FAM189A2 expression was downregulated in tumor tissues, and low expression of FAM189A2 was related to poor survival prognosis (13). In addition, FAM189A2 was also found to be downregulated in endometrial carcinoma, and decreased expression of FAM189A2 was associated with a poor overall survival (14). However, whether the expression of FAM189A1 and FAM189A2 was dysregulated in HCC was still largely unknown. The effect of FAM189A1 and FAM189A2 on the survival of HCC patients has not been investigated.

FAM189B, also known as COTE1 or C1orf2, is located on chromosome 1q22, a locus near the gene for the lysosomal enzyme glucosylceramidase, a deficiency of which has been linked to Gaucher disease (15). FAM189B encodes a membrane protein that is widely expressed in multiple tissues, including heart, brain, placenta, lung, liver, skeletal muscle, kidney, and pancreas(15, 16). Pervious study showed that the expression of FAM189B was upregulated in gastric cancer, and highly expressed FAM189B was associated with a poor overall survival of GC patients (17). In addition, two studies from the same team showed that the expression of FAM189B was also upregulated in HCC, and intervention of FAM189B expression affected the invasion, cell proliferation and colony formation of HCC cell lines (18, 19). However, except for tumor size and Edmondson grade, high expression of FAM189B was not observed to be correlated with other clinical characteristics of the patients in these studies. The effect of FAM189B on the prognosis of HCC patients has not been reported, and the relationship between FAM189B and the clinical and molecular characteristics of HCC remains largely unknown. Further, the aberrant expression of FAM189B and its mechanism of promoting HCC oncogenesis still need to be explored.

In this study, we comprehensively analyzed the expression and characteristics of FAM189 family genes in HCC from multiple datasets through bioinformatics methods. As a result, high expression of FAM189B was found in HCC and associated with unfavorable prognosis of HCC patients. The immunohistochemical assay also showed that the IHC score of FAM189B was higher in tumor tissues than in adjacent tissues. Copy number variation contributed to increased expression of FAM189B in HCC. Highly expressed FAM189B was associated with high AFP level, and TP53 mutation of HCC patients. Gene set enrichment analysis (GSEA) showed that pathways and biological processes related to highly expressed FAM189B were mainly associated with cell proliferation and cell cycle. This study provides a detailed analysis of FAM189B in HCC, which will help us in the understanding the molecular mechanism for the pathogenesis of HCC.

## Materials and methods

### Data acquisition and analysis

The gene expression data and clinical information of HCC patients of TCGA-LIHC (20) cohort were downloaded from UCSC Xena (https://xenabrowser.net/datapages/) database. For the TCGA-LIHC cohort, gene expression data in the format of log_2_(FPKM+1) was downloaded from UCSC Xena and could be directly used in subsequent analysis. The gene expression data and clinical information of HCC patients of ICGC-LIRI-JP (International Cancer Genome Consortium, Liver Cancer-RIKEN, Japan) cohort, which was numbered as HCCDB18, were downloaded from HCCDB database (http://lifeome.net/database/hccdb/home.html). The downloaded gene expression data for both these cohorts were obtained based on RNA-seq technology, and were ready to be used in further bioinformatics analysis. A total of 377 HCC patients from the TCGA-LIHC cohort and 260 HCC patients from ICGC-LIRI-JP cohort were enrolled in this study. The main clinical information of these two cohorts was summarized in Table 1. Another four independent datasets containing HCC tumor and adjacent tissues were downloaded from the GEO (Gene Expression Omnibus) database (https://www.ncbi.nlm.nih.gov/geo/), including GSE14520 (21) (https://www.ncbi.nlm.nih.gov/geo/query/acc.cgi?acc=GSE14520), GSE22058 (22) (https://www.ncbi.nlm.nih.gov/geo/query/acc.cgi?acc=GSE22058), GSE25097(23) (https://www.ncbi.nlm.nih.gov/geo/query/acc.cgi?acc=GSE25097), and GSE50579 (24) (https://www.ncbi.nlm.nih.gov/geo/query/acc.cgi?acc=GSE50579). The preprocessed and normalized gene expression matrix files for these datasets were directly downloaded from the GEO database. Cancer Cell Line Encyclopedia (CCLE) database (https://sites.broadinstitute.org/ccle) is a compilation of gene expression, chromosomal copy number and massively parallel sequencing data from 947 human cancer cell lines (25). In this study, the gene expression and copy number variation data of 24 kinds of HCC cell lines were downloaded from the CCLE database, including JHH6, SUN398, SUN886, SKHEP1, HLF, SNU449, SNU475, LI7, HUH1, JHH4, SNU387, HUH7, SNU382, SNU423, SNU761, JHH2, JHH1, HEP3B, HUH6, SNU878, JHH5, HEPG2, JHH7, PLCPRF5. The correlation between FAM189B gene expression and copy number variation was analyzed in these 24 kinds of HCC cell lines.

**TABLE 1:** Clinical characteristics of patients from TCGA-LIHC, ICGC-LIRI-JP and GSE14520 cohorts.

Clinical characteristics		Total	%
TCGA-LIHC		377	
Age	≤60	180	47.75
	>60	196	51.99
Gender	Female	122	32.36
	Male	255	67.64
Stage	Stage 1	175	46.42
	Stage 2	87	23.08
	Stage 3	86	22.81
	Stage 4	5	1.33
Grade	Grade 1	55	14.59
	Grade 2	180	47.75
	Grade 3	124	32.89
	Grade 4	13	3.45
T classification	T1	185	49.07
	T2	95	25.20
	T3	81	21.49
	T4	13	3.45
N classification	N0	257	68.17
	N1	4	1.06
	NX	115	30.50
M classification	M0	272	72.15
	M1	4	1.06
	MX	101	26.79
Survival status	Alive	244	64.72
	Death	132	35.01
ICGC-LIRI-JP		260	
Age	≤60	55	21.15
	>60	205	78.85
Gender	Female	68	26.15
	Male	192	73.85
Stage	Stage 1	40	15.38
	Stage 2	117	45.00
	Stage 3	80	30.77
	Stage 4	23	8.85
Survival status	Alive	214	82.31
	Death	46	17.69
GSE14520		225	
Age	≤ 60	40	17.78
	>60	181	80.44
Gender	Female	30	13.33
	Male	191	84.89
Predicted risk metastasis signature	Low	114	50.67
	High	107	47.56
ALT	≤ 50 U/L	91	40.44
	>50 U/L	130	57.78
Tumor size	≤ 5 cm	140	62.22
	>5 cm	80	35.56
Multinodular	No	176	78.22
	Yes	45	20.00
Cirrhosis	No	18	8.00
	Yes	203	90.22
TNM stage	Stage 1	93	41.33
	Stage 2	77	34.22
	Stage 3	49	21.78
AFP	Low	118	52.44
	High	100	44.44
Survival status	Alive	136	60.44
	Death	85	37.78

### Gene set enrichment analysis and verification

GSEA is a computational approach that analyze significantly enriched gene groups (26). In this study, GSEA was applied to explore potential mechanism underlying the expression of FAM189B in the pathogenesis of HCC. The TCGA-LIHC samples were divided into FAM189B high and low expression groups based on the median value of FAM189B mRNA expression. The “h.all.v7.5.symbols.gmt” gene sets from the Molecular Signatures Database (MSigDB) were analyzed through GSEA 4.1.0 software. The number of permutations for the analysis was set as 1000. The normalized enrichment score (NES), nominal p value (NOM p), and false discovery rate q value (FDR q) were selected to classify the signaling pathways and biological processes enriched. Gene sets with NES >1, NOM *p* < 0.05, and FDR *q* < 0.25 were regarded as significantly enriched. In addition, the GSEA results related to FAM189B expression were further validated in ICGC-LIRI-JP and GSE14520 datasets, respectively.

### Immunohistochemical assay

Forty-seven pairs of HCC tumor and adjacent tissues were collected for the IHC assay. Clinical information of samples used for IHC assay is presented in Supplementary Table S1. All these samples were obtained from resected tumors. Informed consent was obtained before the study from all patients. This study was approved by the Medical Ethics Committee of Shandong Provincial Qianfoshan Hospital according to the Declaration of Helsinki, and the number of the approval was S092. Immunohistochemical assay was performed according to a routine protocol as previously reported (27). The primary antibody of FAM189B (Invitrogen, PA5-52318) was diluted with the ratio of 1:50. The secondary antibody kit (rabbit two-step detection kit, PV-9001) was purchased from ZSBG-Bio company. The slides were deparaffinized and rehydrated. Antigen retrieval was performed in citrate buffer with high-pressure steam for 3 min. The endogenous peroxidase activity was blocked with 3.0% hydrogen peroxide for 15 min. The sections were incubated with the primary antibody of FAM189B, and then secondary antibody (horseradish peroxidase conjugated, goat anti rabbit antibody) for 60 min, at room temperature. The slide was subsequently incubated in DAB, counterstained with hematoxylin, and mounted. Each sample was scored according to staining intensity and the proportion of stained cells (28). Briefly, the staining intensity was scored as below: no staining = 0, weak staining = 1, moderate staining = 2, and strong staining = 3. The proportion of stained cells was scored as: 0% = 0, 1%–25% = 1, 26%–50% = 2, 51%–75% = 3, and 76%–100% = 4. Final IHC scores were determined by multiplying the staining intensity score and the score of the proportion of stained cells.

### Statistical analysis

R language and GraphPad Prism 8.0 were applied for statistical analysis and graph plot, respectively. The relationship between FAM189B expression and clinical characteristics including cancer stage, histological grade, AFP level, genetic mutation, and predicted risk metastasis signature, was examined using Student’s t test. Univariate and multivariate Cox regression analysis was performed to evaluate the prognostic value of FAM189B in HCC. The degree of correlation between FAM189B expression and copy number variation was examined by Spearman correlation analysis. Time-dependent receiver operating characteristic (ROC) curve analysis was performed to evaluate the prediction precision of FAM189B for overall survival (OS), disease-specific survival (DSS) and progression-free interval (PFI), and the area under curve value (AUC) was calculated. In all analysis, p value < 0.05 was considered statistically significant.

## Results

### Clinical characteristics of the patients included in this study

Table 1 showed the detailed clinical characteristics of patients from TCGA-LIHC and ICGC-LIRI-JP cohorts that were included in the current study. Generally, there were 377 patients from TCGA-LIHC cohort and 260 patients from ICGC-LIRI-JP cohort, respectively. There were 255 males in the LIHC cohort and 192 males in LIRI-JP cohort. The counts of patients with stage 1, 2, 3, and 4 were 175, 87, 86, and 5 in the TCGA-LIHC and 40, 117, 80, and 23 in the ICGC-LIRI-JP cohorts, respectively.

### FAM189B is highly expressed in HCC

**FIGURE 1:**
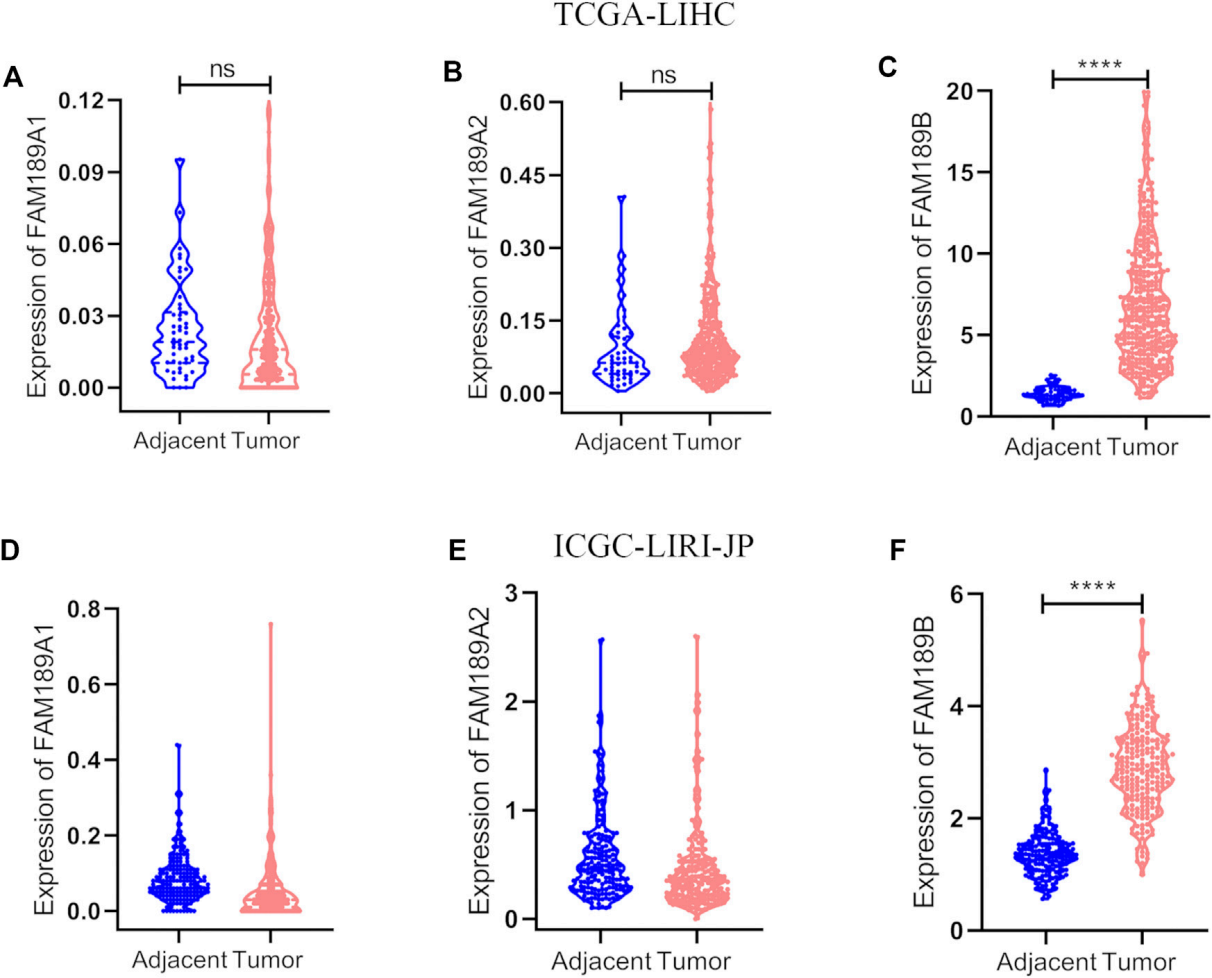
The expression of FAM189 family members in TCGA-LIHC and ICGC-LIRI-JP cohorts. **(A–C)** The expression of FAM189A1 **(A)**, FAM189A2 **(B)**, and FAM189B **(C)** between tumor and adjacent tissues in TCGA-LIHC cohort. **(D–F)** The expression of FAM189A1 **(D)**, FAM189A2 **(E)**, FAM189B **(F)** between tumor and adjacent tissues in ICGC-LIRI-JP cohort. ****, *p* < 0.0001; ns, not significant.

**FIGURE 2:**
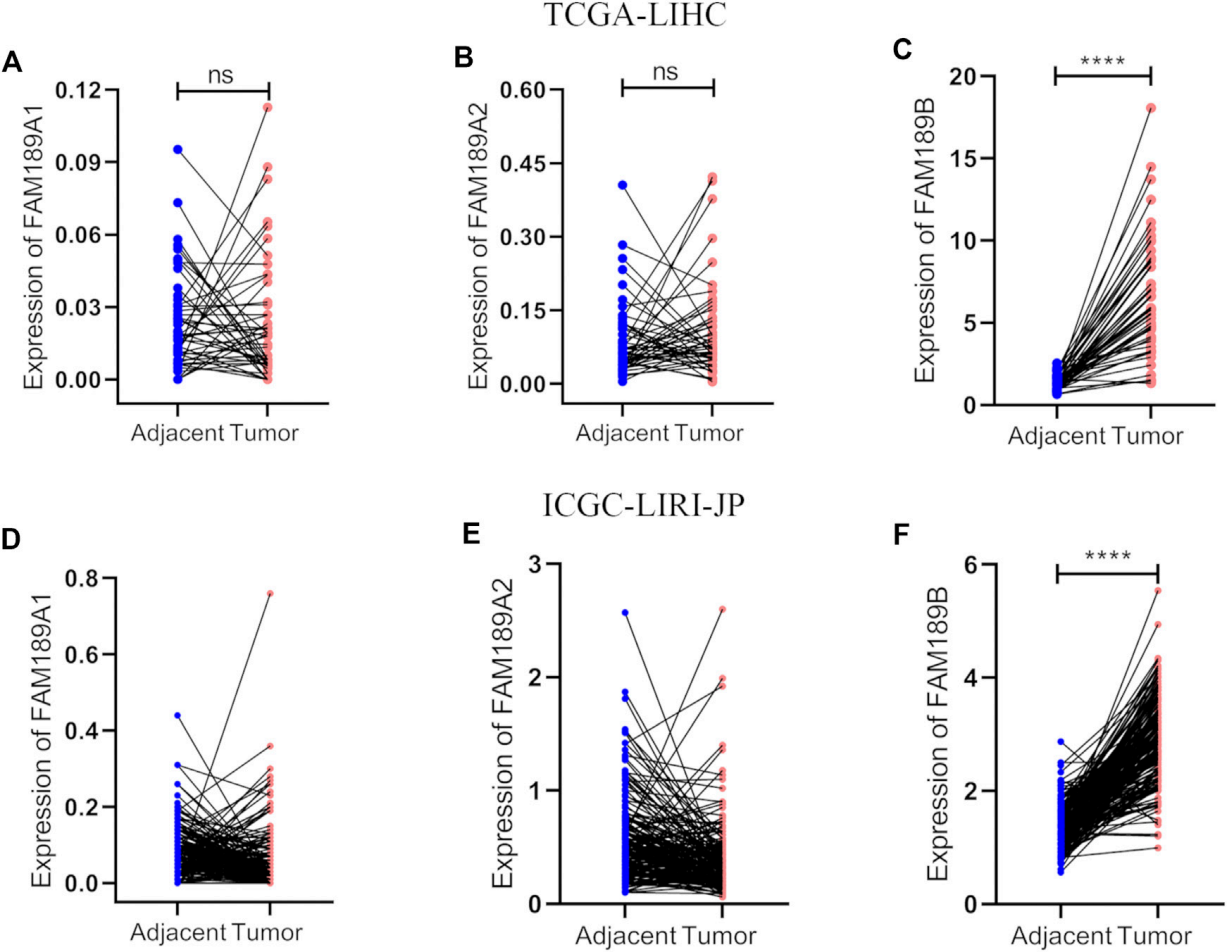
Paired analysis of FAM189 family members in TCGA-LIHC and ICGC-LIRI-JP cohorts. **(A–C)** Paired analysis of the expression of FAM189A1 **(A)**, FAM189A2 **(B)**, FAM189B **(C)** in tumor and matched adjacent tissues in TCGA-LIHC cohort. **(D–F)** Paired analysis of the expression of FAM189A1 **(D)**, FAM189A2 **(E)**, FAM189B **(F)** in tumor and matched adjacent tissues in ICGC-LIRI-JP cohort. ****, *p* < 0.0001; ns, not significant.

**FIGURE 3:**
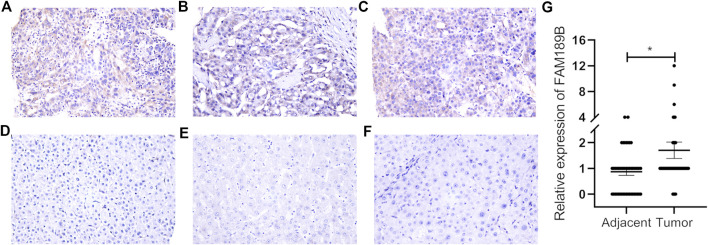
The protein expression level of FAM189B in HCC tissues. **(A–C)** IHC images from three cases of HCC tumor tissues, with the IHC score of 12, 2, 6, respectively. **(D–F)** IHC images from three cases of HCC adjacent tissues, with the IHC score of 0. Case1 **(A,D)**; case2 **(B,E)**; case 3 **(C,F)**. **(G)** Difference in the staining score of FAM189B between adjacent and tumor tissues. *, *p* < 0.05.

We first analyzed the expression of FAM189 family genes in liver cancer patients from TCGA-LIHC cohort. As shown in Figures 1A,B, there was no significant difference for the expression of FAM189A1 and FAM189A2 between HCC tumor and adjacent tissues. However, transcription of FAM189B was significantly upregulated in HCC tumor tissues compared to that in adjacent tissues (Figure 1C). We further validated the expression of FAM189 family genes in ICGC-LIRI-JP cohort. As expected, the expression of FAM189A1 and FAM189A2 between HCC tumor and adjacent tissues did not change significantly (Figures 1D,E), while the expression of FAM189B was significantly upregulated in HCC tumor tissues (Figure 1F). Furthermore, paired analysis showed that the expression of FAM189B in tumor tissues was significantly higher than in matched adjacent tissues (Figure 2C), while no significant difference for FAM189A1 and FAM189A2 was observed between tumor and matched adjacent tissues (Figures 2A,B). The same performance was shown in paired analysis of ICGC-LIRI-JP cohort. As shown in Figures 2D–F, FAM189B was highly expressed in tumor tissues compared to that in matched adjacent tissues, while no significant difference was observed in the expression of FAM189A1 and FAM189A2. Similar results were obtained in multiple independent GEO datasets. As shown in Supplementary Figure S1, FAM189B was highly expressed in HCC tumor tissues compared with that in adjacent tissues in a variety of independent datasets, including GSE14520, GSE22058, GSE25097, and GSE50579. Furthermore, we examined the expression of FAM189B at the protein level in resected adjacent and tumor tissues from HCC patients by IHC assay. As shown in Figures 3A–F, the expression level of FAM189B protein was lower in adjacent tissues, but higher in corresponding tumor tissues. Statistical analysis also showed that the IHC score of FAM189B in tumor tissues was higher than that in adjacent tissues (Figure 3G). Collectively, all these results suggested that the expression level of FAM189B in HCC tumor tissues was significantly increased compared with adjacent normal tissues.

**FIGURE 4:**
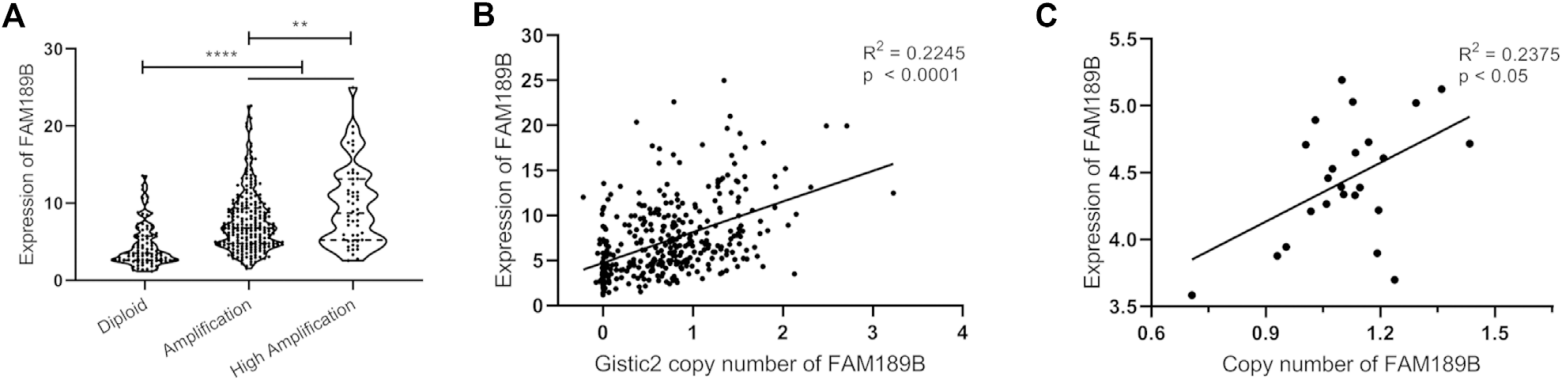
The association of FAM189B mRNA expression and copy number variation in HCC. **(A)** The expression of FAM189B mRNA among diploid, amplification, and high amplification groups. **(B)** Correlation of FAM189B mRNA expression and copy number values in HCC patients. **(C)** Correlation of FAM189B mRNA expression and copy number values in 24 kinds of HCC cell line in CCLE database. **, *p* < 0.01; ****, *p* < 0.0001.

Gene level copy number variation (CNV) is frequently observed and involved in abnormal mRNA transcription in liver cancer (29, 30). Next, we investigated gene level copy number variation of FAM189B and its contribution to increased FAM189B expression in HCC. As shown in Figure 4A, the expression of FAM189B was significantly higher in the amplification group than in diploid group, and further elevated in high amplification group. In addition, the aberrant expression of FAM189B was significantly correlated with elevated copy number values (Figure 4B). This result suggested that copy number variation contributed to increased FAM189B expression in liver cancer tissues. We further validated the contribution of copy number variation to the increased expression of FAM189B in 24 kinds of HCC cell lines from CCLE database. The result showed that the upregulated expression of FAM189B was also significantly correlated with its increased copy number values in these HCC cell lines (Figure 4C). Collectively, results from HCC tumor tissues and cell lines consistently suggested that copy number variation promoted the upregulation of FAM189B mRNA expression levels.

### Highly expressed FAM189B is associated with poor prognosis of HCC patients

**FIGURE 5:**
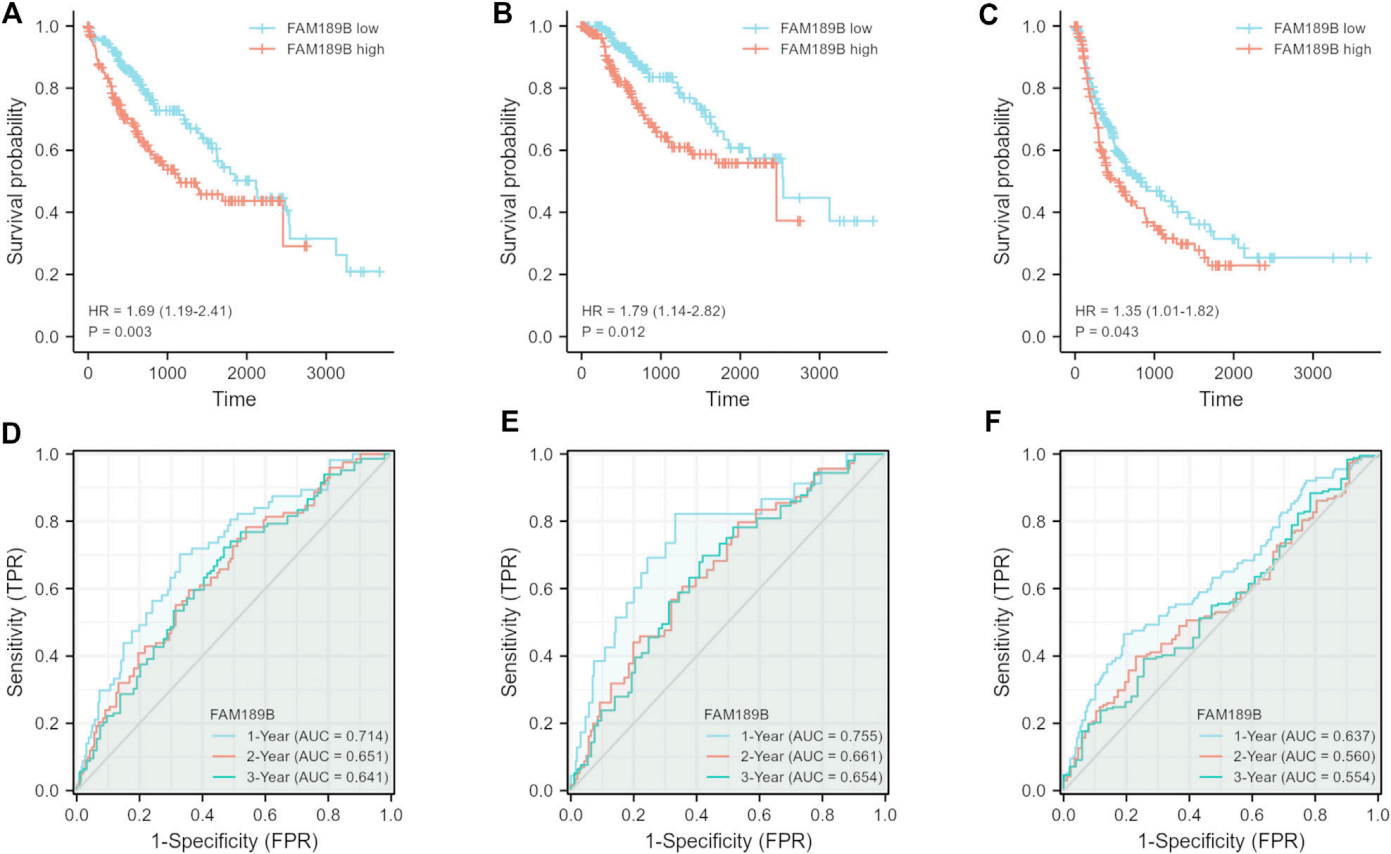
Relationship of FAM189B expression and the prognosis of HCC patients. **(A–C)** Relationship of FAM189B expression and OS **(A)**, DSS **(B)**, and PFI **(C)** of HCC patients in TCGA-LIHC cohort. **(D–F)** Time-dependent ROC curve analysis of the expression of FAM189B on OS **(D)**, DSS **(E)**, PFI **(F)** of HCC patients in TCGA-LIHC cohort.

We continued to study the effect of FAM189 family genes on the prognosis of HCC patients. Total TCGA-LIHC patients were separated into two groups according to the median transcription value of FAM189 family genes. The effect of FAM189B expression on survival outcome of HCC patients, including OS, DSS, PFI, are shown in Figure 5. High FAM189B expression group had significantly unfavourable OS (HR = 1.69, 95%CI = 1.19–2.41, *p* = 0.003, Figure 5A), DSS (HR = 1.79, 95%CI = 1.14–2.82, *p* = 0.012, Figure 5B), and PFI (HR = 1.35, 95%CI = 1.01–1.82, *p* = 0.043, Figure 5C), compared with the low expression group. Time-dependent ROC curve analysis showed that FAM189B had certain prediction abilities in predicting survival outcome (Figures 5D–F). However, both the expression of FAM189A1 and FAM189A2 had no significant effect on the survival of HCC patients (Supplementary Figure S2). We further validated the prognostic ability of FAM189B in another two datasets, including ICGC-LIRI-JP and GSE14520. Highly expressed FAM189B is correlated with a poor survival for HCC patients in both ICGC-LIRI-JP (HR = 2.23, 95%CI = 1.17–4.24, *p* = 0.014, Supplementary Figure S3A) and GSE14520 (HR = 1.59, 95%CI = 1.03–2.46, *p* = 0.037, Supplementary Figure S3B) datasets. Collectively, these results suggested that high expression of FAM189B predicted a poor prognosis for HCC patients, and FAM189B may be a novel prognostic indicator for HCC. Furthermore, we assessed the prognostic value of FAM189B through Cox regression analysis based on the RNA expression data and clinical characteristics of HCC patients. As shown in Table 2, the results showed that high expression of FAM189B was associated with unfavourable OS and DSS for HCC patients. Cox regression analysis in GSE14520 dataset also confirmed the prognostic value of FAM189B in HCC (Supplementary Table S2). Collectively, FAM189B was confirmed to be a potential prognostic factor for HCC patients.

**TABLE 2:** Cox regression analysis of clinical characteristics plus FAM189B for OS and DSS of TCGA-LIHC patients.

Characteristics	OS				DSS			
	Univariate		Multivariate		Univariate		Multivariate	
	HR (95%CI)	p value	HR (95%CI)	p value	HR (95%CI)	p value	HR (95%CI)	p value
Age (>60 vs. ≤ 60)	1.205 (0.850–1.708)	0.295			0.846 (0.543–1.317)	0.458		
Gender (Male vs. Female)	0.793 (0.557–1.130)	0.200			0.813 (0.516–1.281)	0.373		
AFP(ng/ml) (>400 vs. ≤ 400)	1.075 (0.658–1.759)	0.772			0.867 (0.450–1.668)	0.668		
Histologic grade (G3 & G4 vs. G1 & G2)	1.091 (0.761–1.564)	0.636			1.086 (0.683–1.728)	0.726		
**Pathologic stage (Stage III and IV vs. Stage Iand II)**	**2.504 (1.727–3.631)**	**<0.001**	**2.547 (1.755–3.697)**	**<0.001**	**3.803 (2.342–6.176)**	**<0.001**	**3.979 (2.445–6.478)**	**<0.001**
Vascular invasion (Yes vs. No)	1.344 (0.887–2.035)	0.163			1.277 (0.707–2.306)	0.418		
Fibrosis ishak score (3/4&5/6 vs. 0&1/2)	0.740 (0.445–1.232)	0.247			0.660 (0.340–1.279)	0.218		
**FAM189B (High vs. Low)**	**1.662 (1.169–2.365)**	**0.005**	**1.713 (1.176–2.496)**	**0.005**	**1.755 (1.116–2.759)**	**0.015**	**1.985 (1.199–3.287)**	**0.008**

### Relationship of FAM189B and clinical and molecular characteristics of HCC patients

**FIGURE 6:**
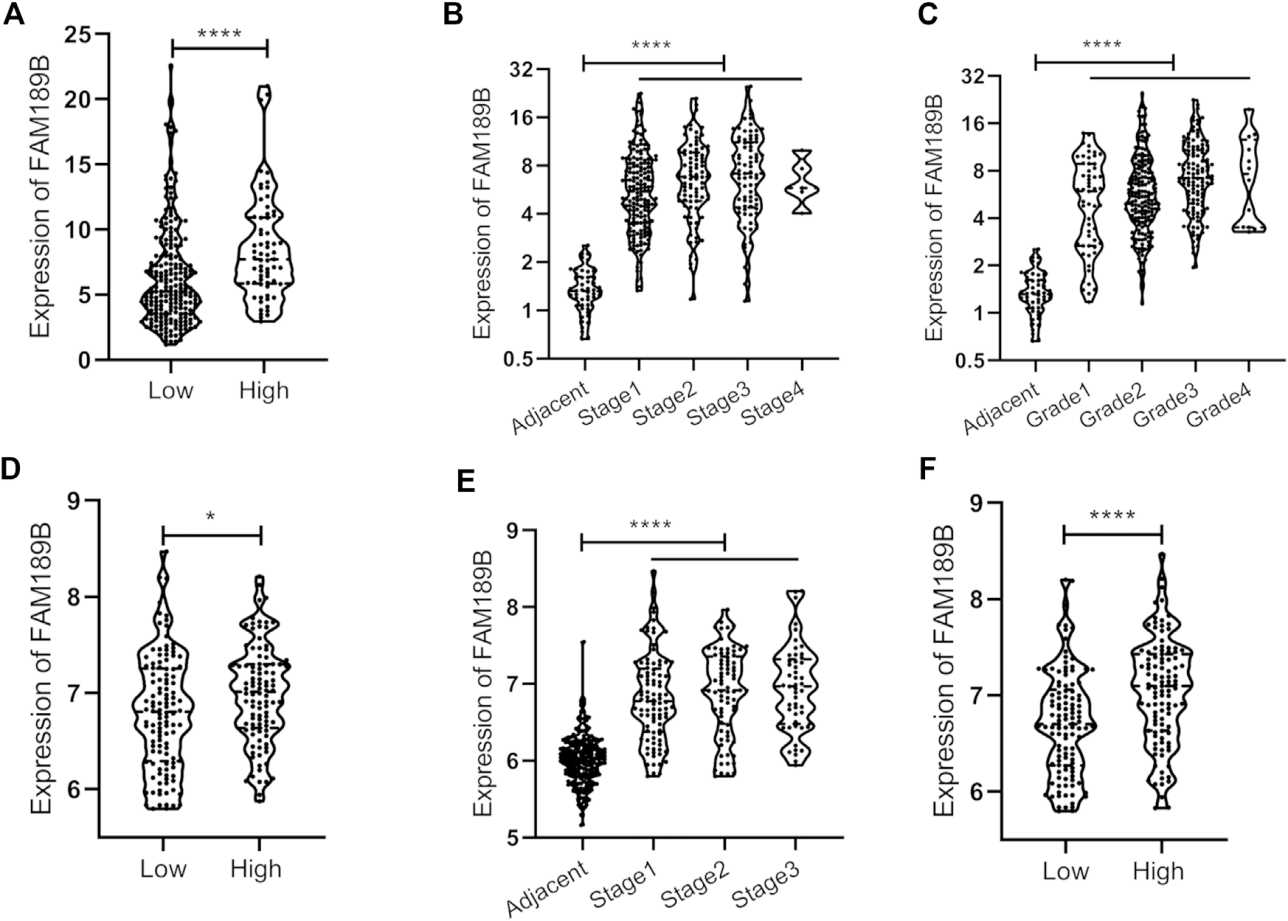
The association between FAM189B expression and the clinical pathological characteristics of HCC patients. **(A**, **B** **and** **C** **)**The relationship of FAM189B expression with AFP level **(A)**, cancer stage **(B)**, and histological grade **(C)** in HCC patients from TCGA-LIHC cohort. **(D–F)**The relationship of FAM189B expression with AFP level **(D)**, cancer stage **(E)**, and predicted risk metastasis signature **(F)** in HCC patients from GSE14520 cohort. *, *p* < 0.05; ****, *p* < 0.0001.

**FIGURE 7:**
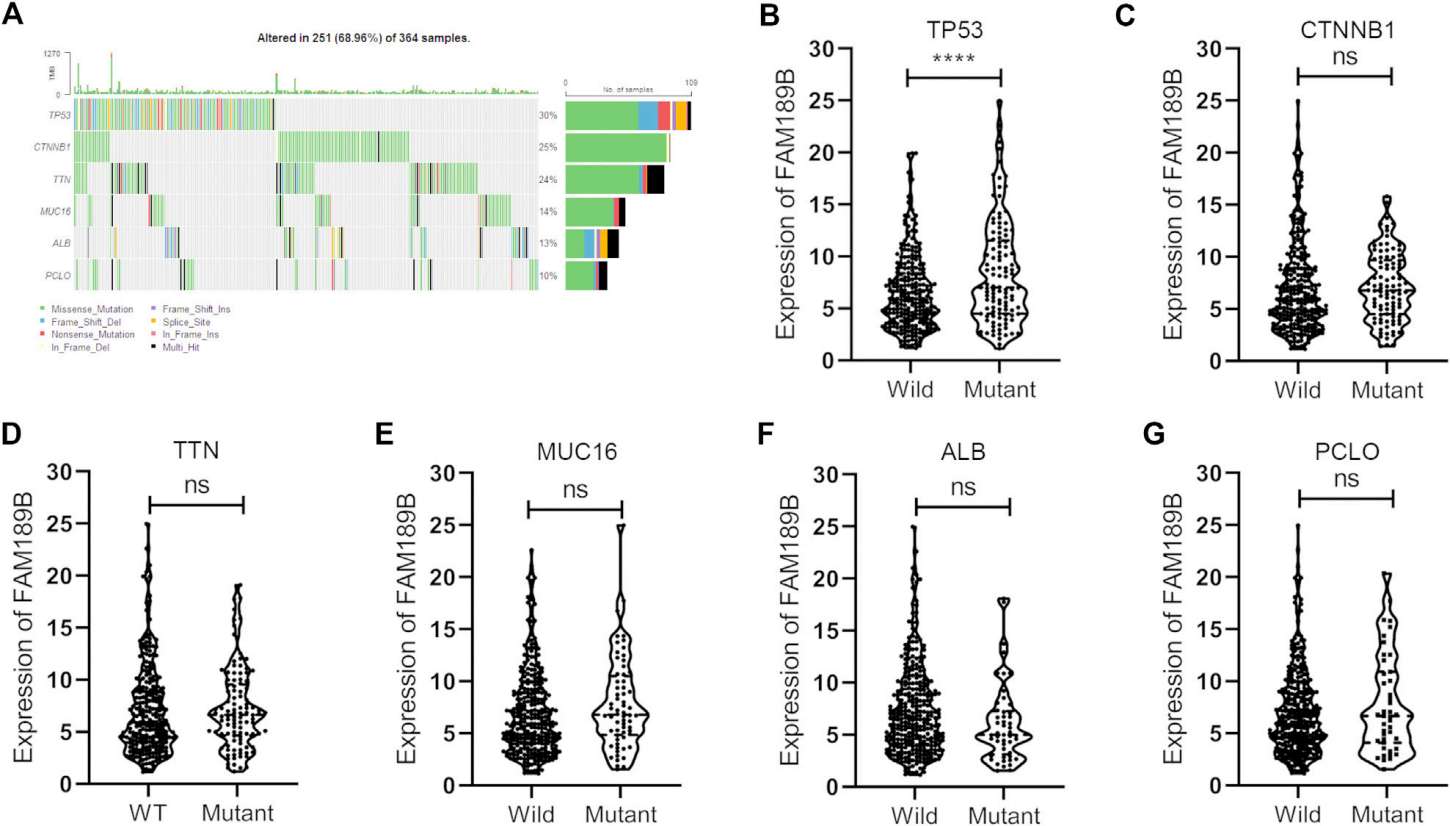
The association between FAM189B expression and the molecular characters of HCC patients. **(A)** Waterfall plot of genetic mutation and top 6 mutated genes of HCC patients from TCGA-LIHC cohort. **(B–G)**The relationship of FAM189B expression with the mutation of top 6 mutated genes, including TP53 **(B)**, CTNNB1 **(C)**, TTN **(D)**, MUC16 **(E)**, ALB **(F)**, PCLO **(G)**. ****, *p* < 0.0001; ns, not significant.

We further analyzed the relationship of FAM189B expression and clinical and molecular characteristics of HCC patients. As shown in Figure 6, results from the analysis of TCGA-LIHC cohort showed that FAM189B was highly expressed in high AFP group compared with that in low group (Figure 6A). However, there was no significant association between FAM189B expression and cancer stage or tumor grade of HCC patients (Figures 6B,C).We further analyzed the relationship of FAM189B and clinical characteristics of HCC patients in GSE14520 dataset. Consistently, high expression of FAM189B was closely associated with high AFP level (Figure 6D), while there was no significant association between FAM189B expression and stage of HCC (Figure 6E). Interestingly, patients with high predicted risk metastasis signature (21, 31), which is predictive of the risk of HCC recurrence and survival, tended to express high level of FAM189B (Figure 6F). Furthermore, genetic mutation analysis was performed in HCC patients. As shown in Figure 7A, TP53 (30%), CTNNB1 (25%), TTN (24%), MUC16 (14%), ALB (13%), and PCLO (10%) ranked the top 6 mutated genes, mounting for 251 (68.96%) of total 364 samples. This result is consistent with a previous report (32). Higher levels of FAM189B mRNA tended to be expressed in TP53 mutant tissues from HCC patients (Figure 7B). There was no significant difference for the expression of FAM189B between wild and mutant groups of CTNNB1 (Figure 7C), TTN (Figure 7D), MUC16 (Figure 7E), ALB (Figure 7F), and PCLO (Figure 7G). Collectively, these results revealed that high expression of FAM189B was associated with high AFP level and TP53 mutation, while there was no significant association between FAM189B and stage or grade of HCC.

### GSEA analysis of FAM189B in HCC

**FIGURE 8:**
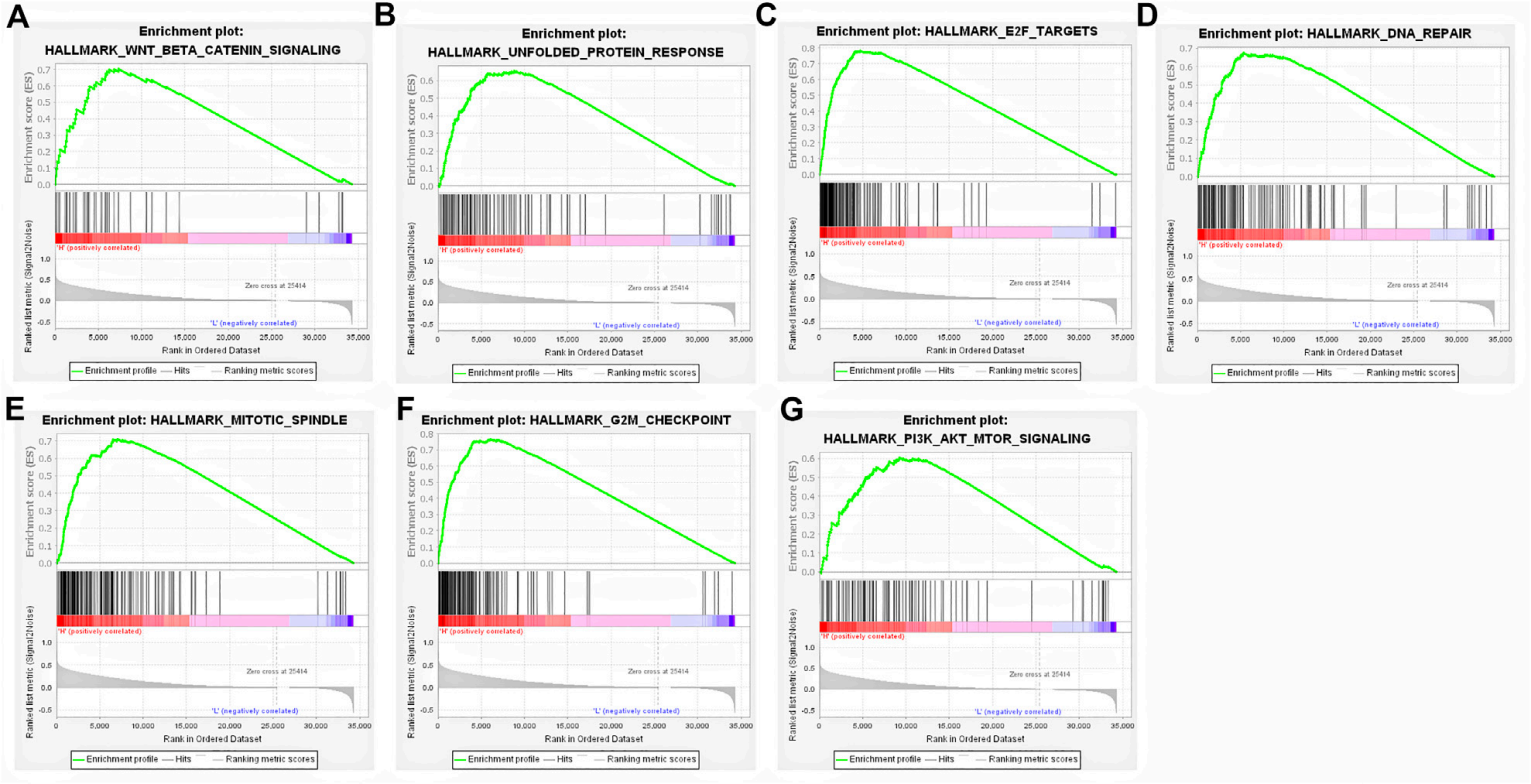
Signal pathways and biological processes associated with highly expressed FAM189B determined by GSEA. **(A)** WNT/β-catenin signaling, **(B)** Unfolded protein response, **(C)** E2F targets, **(D)** DNA repair, **(E)** mitotic spindle, **(F)** G2M checkpoint, **(G)** PI3K/Akt/mTORsignaling.

To further identify the potential signal pathways and biological processes associated with highly expressed FAM189B in HCC, the GSEA analysis was implemented in TCGA-LIHC cohort and further validated in ICGC-LIRI-JP and GSE14520 cohorts. As shown in Table 3, multiple signal pathways and biological processes related to highly expressed FAM189B were enriched in these three datasets. Further, many common signal pathways and biological processes related to FAM189B were enriched in these three datasets, including WNT-β-catenin signaling (Figure 8A), unfolded protein response (Figure 8B), E2F targets (Figure 8C), DNA repair (Figure 8D), mitotic spindle (Figure 8E), G2M checkpoint (Figure 8F) and PI3K-AKT-mTOR signaling (Figure 8G). All these signal pathways and biological processes were involved in the occurrence and progression of HCC. These results indicated that signal pathways and biological processes related to highly expressed FAM189B may play an important role in the oncogenesis of HCC.

**TABLE 3:** Signal pathways and biological processes associated with FAM189B determined by GSEA in TCGA-LIHC and ICGC-LIRI-JP cohorts.

Description	NES	NOM p-val	FDR q-val
TCGA-LIHC			
HALLMARK_WNT_BETA_CATENIN_SIGNALING	1.673	0.000	0.059
HALLMARK_UNFOLDED_PROTEIN_RESPONSE	1.645	0.002	0.043
HALLMARK_E2F_TARGETS	1.636	0.000	0.035
HALLMARK_DNA_REPAIR	1.636	0.000	0.026
HALLMARK_MITOTIC_SPINDLE	1.626	0.000	0.023
HALLMARK_G2M_CHECKPOINT	1.623	0.000	0.020
HALLMARK_MYC_TARGETS_V1	1.588	0.002	0.029
HALLMARK_MYC_TARGETS_V2	1.539	0.015	0.049
HALLMARK_PI3K_AKT_MTOR_SIGNALING	1.513	0.010	0.059
ICGC-LIRI-JP			
HALLMARK_MITOTIC_SPINDLE	1.936	0.000	0.063
HALLMARK_DNA_REPAIR	1.924	0.000	0.039
HALLMARK_G2M_CHECKPOINT	1.834	0.010	0.065
HALLMARK_PI3K_AKT_MTOR_SIGNALING	1.735	0.010	0.116
HALLMARK_E2F_TARGETS	1.734	0.024	0.095
HALLMARK_WNT_BETA_CATENIN_SIGNALING	1.693	0.008	0.111
HALLMARK_UNFOLDED_PROTEIN_RESPONSE	1.616	0.025	0.161
HALLMARK_HEME_METABOLISM	1.556	0.012	0.166
GSE14520			
HALLMARK_WNT_BETA_CATENIN_SIGNALING	1.871	0.002	0.013
HALLMARK_MYC_TARGETS_V1	1.838	0.011	0.012
HALLMARK_MITOTIC_SPINDLE	1.717	0.004	0.037
HALLMARK_G2M_CHECKPOINT	1.698	0.023	0.037
HALLMARK_E2F_TARGETS	1.680	0.025	0.034
HALLMARK_UNFOLDED_PROTEIN_RESPONSE	1.666	0.004	0.032
HALLMARK_DNA_REPAIR	1.581	0.036	0.061

## Discussion

In this study, we used genome-wide expression and clinical data from multiple independent datasets to analyze the expression and clinical significance of FAM189 family genes in HCC and identified that FAM189B was consistently highly expressed in tumor tissue and closely associated with poor prognosis of HCC patients. The signal pathways and biological processes related to FAM189B were mainly associated with cell proliferation and cell cycle regulation. All these results suggested that FAM189B was a potential prognostic biomarker for HCC, and FAM189B could be a potential therapeutic target for HCC.

HCC is one of the most prevalent cancers, causing more than 500,000 deaths every year in the world (2). Many studies have shown that metastasis at advanced stage is one of the leading causes of HCC-related death, highlighting the importance of early diagnosis and effective treatment of the disease (33). Although numerous advances in HCC diagnosis and treatment have been achieved, only a small percentage of HCC patients are detected at an early stage, and the prognosis of HCC patients remains unsatisfactory (34). Therefore, investigating effective prognostic biomarkers and potential therapeutic targets for HCC are still urgently needed.

In this study, we first examined the transcription level of FAM189 family genes in HCC in TCGA-LIHC and ICGC-LIRI-JP cohorts respectively. FAM189B was highly expressed in HCC tumor tissues compared with adjacent tissues, while the expression of FAM189A1 and FAM189A2 was not significantly changed. Paired analysis confirmed that FAM189B expression in HCC tumor tissues was higher than that in matched adjacent tissues. Further, upregulated expression of FAM189B in HCC was validated in multiple independent GEO datasets. Wu et al. recently analyzed a number of gastric cancer datasets, and reported that expression of FAM189B was upregulated in gastric tumor tissues, and predicted a poor prognosis for gastric cancer patients (17). Additionally, two previous studies reported that mRNA of FAM189B was upregulated in HCC specimens (18, 19). In the current study, high expression of FAM189B was identified through a variety of independent datasets. Furthermore, the expression of FAM189B at the protein level in HCC was detected by IHC assay. The IHC score of FAM189B was elevated in tumor tissues compared with that in adjacent tissues, although this result of the expression changes of FAM189B at the protein level needs to be verified in more samples in the future study. In addition, CNV analysis revealed that increased expression of FAM189B was significantly correlated with elevated copy number values, indicating that CNV contributed to increased FAM189B expression in HCC.

Subsequently, we investigated the prognostic value of FAM189 family members in HCC and further validated in multiple independent datasets. FAM189A1 and FAM189A2 had no significant effect on the survival of HCC patients. However, highly expressed FAM189B was significantly associated with poor OS, DSS, and PFI for HCC patients. The unfavorable effect of FAM189B on the survival outcome of HCC patients was further confirmed in ICGC-LIRI-JP and GSE14520 datasets. To our knowledge, this is the first report that high expression of FAM189B predicts a poor prognosis for liver cancer patients. Univariate and multivariate Cox regression analysis further confirmed that highly expressed FAM189B was a potential unfavorable prognostic factor for OS and DSS of HCC patients. All the above results suggested that high expression of FAM189B was closely related to poor prognosis of HCC patients.

We further investigated the relationship between FAM189B expression and the clinical and molecular characteristics of HCC patients. FAM189B was highly expressed in high AFP group compared with that in low AFP group. However, there was no significant association between FAM189B and stage or grade of HCC. In addition, interestingly, highly expressed FAM189B was associated with high predicted metastasis signature, indicating that FAM189B is associated with the metastasis of HCC. Furthermore, genetic mutation analysis of the liver cancer patients showed that TP53, CTNNB1, TTN, MUC16, ALB, and PCLO ranked the top 6 mutated genes in HCC, which is consistent with the previous reports (35–37). The expression of FAM189B in TP53-mutant group was higher than that in TP53-wild group. This is the first report of the association between FAM189B expression and TP53 mutation in human cancer. These results suggested that high expression of FAM189B was associated with high AFP level and TP53 mutation, while not associated with stage or grade of HCC. GSEA analysis was performed to further explore the abnormal changes in signal pathways and biological processes related to highly expressed FAM189B in HCC patients. Results from multiple datasets showed that upregulated expression of FAM189B was primarily linked with WNT-β-catenin signaling, unfolded protein response, E2F targets, DNA repair, mitotic spindle, G2M checkpoint and PI3K-AKT-mTOR signaling in HCC. All these enriched signal pathways and biological processes were markedly associated with the occurrence and progression of HCC (38–41), indicating a pivotal role for FAM189B in the pathogenesis of HCC. However, this result should be further verified in future studies.

In conclusion, this study systematically and comprehensively analyzed the expression pattern, prognostic value, and potential mechanism of FAM189B in the occurrence and progression of HCC. These results provide insights to identify novel prognostic and clinical indicators and potential therapeutic targets for HCC, which may help us to more precisely predict the survival and personalize the treatment for HCC patients.

## Data Availability

The datasets presented in this study can be found in online repositories. The names of the repository/repositories and accession number(s) can be found in the article/Supplementary Material.

## Supplementary material

SUPPLEMENTARY FIGURE S1 The expression of FAM189B between tumor and adjacent tissues in multiple GEO datasets. **(A)** GSE14520, **(B)** GSE22058, **(C)** GSE25097, **(D)** GSE50579. ***, *p* < 0.001; ****, *p* < 0.0001.

SUPPLEMENTARY FIGURE S2 The relationship of FAM189A1 and FAM189A2 expression and the prognosis of HCC patients in TCGA-LIHC cohort. **(A–C)** Relationship of FAM189A1 expression with OS **(A)**, DSS **(B)**, and PFI **(C)** of HCC patients. **(D–F)** Relationship of FAM189A2 expression with OS **(D)**, DSS **(E)**, and PFI **(F)** of HCC patients.

SUPPLEMENTARY FIGURE S3 The relationship of FAM189B and the prognosis of HCC patients in ICGC-LIRI-JP and GSE14520 cohorts. **(A)** Relationship of FAM189B and the prognosis in ICGC-LIRI-JP. **(B)** Relationship of FAM189B and the prognosis in GSE14520.
