# Supplementary material for: Highly expressed FAM189B predicts poor prognosis in hepatocellular carcinoma
Source: Pathol Oncol Res. 2022 Nov 25;28:1610674. doi: 10.3389/pore.2022.1610674 (PMC9732019; doi:10.3389/pore.2022.1610674)
Supplement: Supplementary file 4 [file Table2.docx]

Table S2: Cox regression analysis of clinical characteristics plus FAM189B for survival of patients in GSE14520 dataset.

| Characteristics | Survival | | | |
| --- | --- | --- | --- | --- |
|  | Univariate | | Multivariate | |
|  | HR  (95%CI) | P value | HR  (95%CI) | P value |
| Age  (>60 vs <=60) | 0.97  (0.55-1.7) | 0.903 |  |  |
| Gender  (Male vs Female) | 1.7  (0.82-3.5) | 0.153 |  |  |
| Multinodular  (Yes vs No) | 1.6  (0.99-2.6) | 0.057 |  |  |
| **TNM stage**  **(stageⅡ&Ⅲvs stageⅠ)** | **3.5**  **(2.2-5.5)** | **<0.001** | **3.3**  **(2.1-5.3)** | **<0.001** |
| **FAM189B**  **(High vs Low)** | **1.6**  **(1.1-2.4)** | **0.012** | **1.5**  **(1.1-2.2)** | **0.027** |
